# PD-1 Inhibitors Could Improve the Efficacy of Chemotherapy as First-Line Treatment in Biliary Tract Cancers: A Propensity Score Matching Based Analysis

**DOI:** 10.3389/fonc.2021.648068

**Published:** 2021-06-17

**Authors:** Miaomiao Gou, Yong Zhang, Tiee Liu, Haiyan Si, Zhikuan Wang, Huan Yan, Niansong Qian, Guanghai Dai

**Affiliations:** ^1^ Medical Oncology Department, The First Medical Center, Chinese People’s Liberation Army General Hospital, Beijing, China; ^2^ Medical Oncology Department, The Second Medical Center, Chinese People’s Liberation Army General Hospital, Beijing, China; ^3^ The Hainan Medical Center, Chinese People’s Liberation Army General Hospital, Sanya, China

**Keywords:** BTC, PD-1 inhibitor, chemotherapy, immunotherapy, PMS

## Abstract

**Background:**

There are limited treatment options for advanced biliary tract cancers (BTCs), including intrahepatic cholangiocarcinoma, extrahepatic bile duct cancer, gallbladder cancer. We compared the efficacy and safety of PD-1 inhibitors plus chemotherapy and chemotherapy alone as first-line treatment in patients with advanced BTC.

**Methods:**

We retrospectively reviewed patients with BTC treated at the oncology department of the Chinese PLA general hospital receiving PD-1 inhibitor with chemotherapy (anti-PD-1+C group) or chemotherapy alone (C group). Propensity Score Matching (PSM) (1:1) was performed to balance potential baseline confounding factors. Progression-free survival (PFS) was analyzed using Kaplan–Meier survival curves with log-rank tests. Objective response rate (ORR), disease control rate (DCR), and safety were also analyzed.

**Results:**

This study included 75 patients who received PD-1 inhibitors (including Pembrolizumab, Nivolumab, Sintilimab, Toripalimab) plus chemotherapy and 59 patients who received chemotherapy alone. After matching, there were no significant differences between the two groups for baseline characteristics. Within the matched cohort, the median PFS was 5.8m in the anti-PD-1+C group, which was significantly longer than the C group, at 3.2m (HR: 0.47, 95% CI 0.29 to 0.76, P = 0.004). The ORR was 21.7% and DCR was 80.4% in the anti-PD-1+C group, while the ORR was 15.2% and DCR was 69.6% in the C group. No significant differences were found in the ORR and DCR between the two groups (P=0.423, P=0.231). Grade 3 or 4 treatment was related to adverse events (AEs) that occurred in the anti-PD-1+C group, namely hypothyroidism (n=3, 6.5%), rash (n=2, 4.2%), and hepatitis (n=1, 2.2%). There was no AE-related death. The grade 3-4 leukopenia rate was similar in the two groups (4.3% vs. 6.5%).

**Conclusions:**

Anti-PD-1 therapy plus chemotherapy prolonged the PFS compared with chemotherapy alone in advanced BTC with controllable AEs. Further clinical trials are needed to confirm this result.

## Introduction

Biliary tract cancer (BTC) consists of intrahepatic cholangiocarcinoma (iCCA), extrahepatic cholangiocarcinoma (eCCA), and gallbladder cancers (GBC). The 5-year survival rate is 0% (1) due to poor biological behavior. Gemcitabine plus cisplatin (GP) (2) and gemcitabine plus s-1 (GS) (3) are recommended as first-line therapy for advanced BTC in the 2018 version of the National Comprehensive Cancer Network (NCCN) guideline. Although the GS regime is not worse than the GP regime in terms of ORR, DCR, and survival; the overall survival is still dismal. Thus, explorations of more effective treatment combinations are warranted.

Programmed cell death protein -1 (PD-1) inhibitors have shown promising results in many tumor types (4–6). However, few studies of anti-PD-1 therapy in BTC were reported before 2015. In 2015, a study determining the efficacy of Pembrolizumab for patients with mismatch-repair inefficient (MSI) tumors (7), reported that four patients with BTC achieved a partial response to anti-PD-1 monotherapy, representing a new milestone in the treatment of BTC. In 2017, the Keynote-028 study (8) showed that Pembrolizumab monotherapy as second-line or after treatment achieved an ORR of 17% in BTC expressing programmed death ligand-1 (PD-L1). However, data from subsequent studies have shown the limited efficacy of PD-1 inhibitor monotherapy (9, 10). We hypothesized that a PD-1 inhibitor plus chemotherapy could improve the efficacy of anti-PD-1 or chemotherapy monotherapy. Based on this hypothesis, we reviewed patients with advanced BTC treated in our clinic who were administered chemotherapy alone or PD-1 inhibitor plus chemotherapy and compared the efficacy and safety of the two groups.

## Patients and Methods

### Patients and Groups

**Figure 1:**
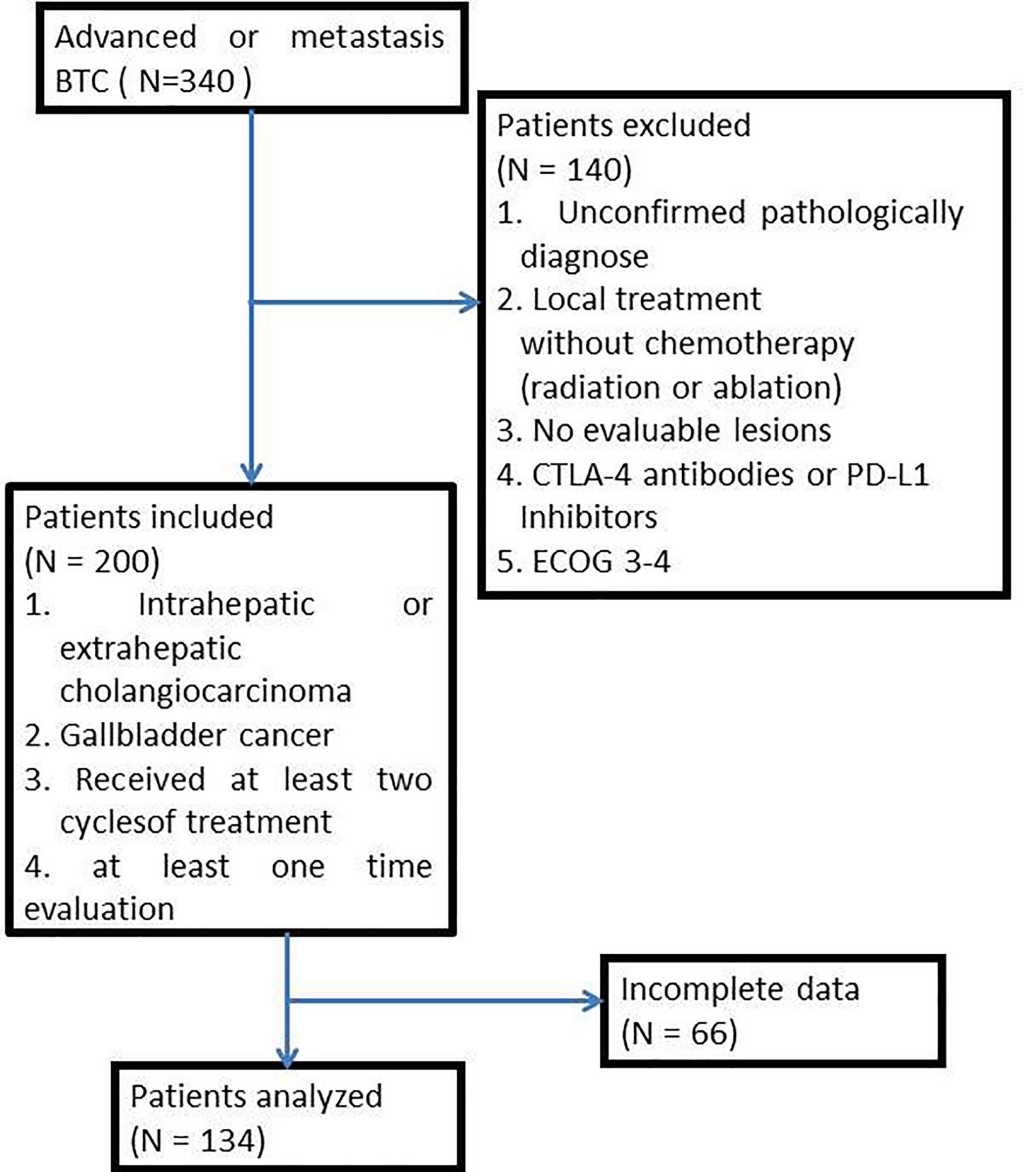
Flow chart of patient inclusion.

From January 2017 to December 2020, 340 patients with advanced BTC were treated in the Chinese People’s Liberation Army General Hospital (PLAGH). Patients were excluded if they (1) had an unconfirmed pathologically diagnosis; (2) received only local treatment (radiation or ablation) without chemotherapy; (3) were without evaluable lesions; (4) received any cytotoxic T-lymphocyte-associated protein 4 (CTLA-4) antibodies or PD-L1 inhibitors; (5) had an ECOG performance status (ECOG PS) score of 3-4. Concerning the quality of data, we included patients who had intrahepatic or extrahepatic cholangiocarcinoma or gallbladder cancer and received at least two cycles of treatment and had finished at least one evaluation of the tumor response in accord to the Response Evaluation Criteria in Solid Tumors (RESIST) criteria (1.1). Complete clinical characters were collected from medical records, including primary tumor location, pathology typing, metastases organ, and treatment regimen (**Figure 1**). This study was approved by the ethics committee of PLAGH, and all patients signed consent for making their data public.

### Treatment and Dose Modification

Chemotherapy regimens involved nab-Paclitaxel-based therapy, gemcitabine-based therapy, platinum-based therapy, were given every three weeks as one cycle. PD-1 inhibitor was administered synchronously with each chemotherapy cycle, and dosage was based on the specific medication, such as Nivolumab 3mg/kg, Pembrolizumab 3mg/kg, Sintilimab 200mg per cycle, or Toripalimab as a stable 240mg dosage. The regimen was based on the patient’s condition and preference. All the patients signed informed consent for treatment.

### Efficacy and Safety Assessments

Patients were selected until the cut-off date of December 1, 2020. Evaluated results were extracted from medical records and verified by radiologists using computed tomography. The primary endpoint for both groups was progression-free survival (PFS, the duration from treatment until progressive disease or death), and the second endpoint was objective response rate (ORR, the percentage of patients with a confirmed complete/partial response) and disease control rate [DCR, percentage of patients with a confirmed complete response (CR)/partial response (PR) and stable disease (SD)]. Adverse events (AEs) were assessed using the Terminology Criteria for Adverse Events, version 4.0, and ESMO Clinical Practice Guidelines for the management of toxicities from immunotherapy (11).

### Statistical Analysis

To control for selection bias and confounding factors, a propensity score matching analysis was performed. The propensity score was estimated multivariate logistic regression according to the following variables: gender, ECOG PS score, primary tumor location, chemotherapy regime, number of metastatic organs. The estimated propensity score matched patients treated with chemotherapy in a ratio of 1:1 to patients treated with chemotherapy plus anti-PD-1 inhibitors, using the nearest-neighbor method within the matching strategy.

Non-parsimonious comparison of rates was performed using the Chi-square test. Progression-free survival was summarized and compared using the Kaplan–Meier method. The hazard ratio (HR) was calculated using Cox proportional hazard regression modeling. Exploratory univariate analyses were performed with the log-rank test for each group. AEs were summarized using percentages and frequency counts. Statistical analysis was performed using SPSS version 22.0 (SPSS Inc., Chicago, IL, USA). P<0.05 was regarded as statistically significant.

## Results

During the study period, 59 patients underwent chemotherapy, and 75 patients were given chemotherapy + PD-1 inhibitor. Baseline clinical features are presented in **Table 1**. Before propensity score matching, there were more female patients in the anti-PD-1 plus chemotherapy group (anti-PD-1+C group) than in the chemotherapy group (C group) (40% vs. 23.7%; P=0.04). In addition, there were more patients with multiple organ metastases (more than 2) in the anti-PD-1+C group than in the C group (44.0% vs. 27.1%, P=0.045). There were more patients without liver metastasis in the anti-PD-1+C group than in the C group (57.3% vs. 32.2%, P=0.004). There were no statistically significant differences at baseline between the two groups for other variables. We did not compare levels of PD-L1 expression because the data of PD-L1 expression was incomplete. Ten patients had PD-L1 expression and 15 were PD-L1-negative in the anti-PD-1+C group; the remaining patients’ status was unknown.

**Table 1:** Baseline characteristics by procedure type before and after propensity score matching.

Characteristics	Before propensity score matching					After propensity score matching				
	C	%	anti-PD-1+C group	%	P	C	%	anti-PD-1+C group	%	P
No. patients	59		75			46		46		
Age median =57 n (%)										
>=57	29	49.2%	39	52.0%	0.744	22	47.8%	22	47.8%	1.000
<57	30	50.8%	36	48.0%		24	52.2%	24	52.2%	
Gender n (%)										
Male	45	76.3%	45	60.0%	0.047	32	69.6%	31	67.4%	0.823
Female	14	23.7%	30	40.0%		14	30.4%	15	32.6%	
*Tumor_location n (%)										
Extrahepatic	27	45.8%	33	44.0%	0.839	22	47.8%	18	39.1%	0.403
Intrahepatic	32	54.2%	42	56.0%		24	52.2%	28	60.9%	
Histological_differentiation n (%)										
Poorly	32	54.2%	33	44.0%	0.311	23	50.0%	24	52.2%	0.841
Moderately	25	42.4%	41	54.7%		22	47.8%	21	45.7%	
Well	2	3.4%	1	1.3%		1	2.2%	1	2.2%	
No.of metastasis organs n (%)										
<=2	43	72.9%	42	56.0%	0.045	32	69.6%	29	63.0%	0.510
>2	16	27.1%	33	44.0%		14	30.4%	17	37.0%	
Liver metastasis n (%)										
No	19	32.2%	43	57.3%	0.004	18	39.1%	21	45.7%	0.529
Yes	40	67.8%	32	42.7%		28	60.9%	25	54.3%	
**ECOG PS n (%)										
0	43	72.9%	47	62.7%	0.130	32	69.6%	34	73.9%	0.650
1	9	15.3%	9	12.0%		7	15.2%	6	13.0%	
>=2	7	11.9%	19	25.3%		7	15.2%	6	13.0%	
Regime n (%)										
nab-Paclitaxel based	40	67.8%	54	72.0%	0.656	34	73.9%	33	71.7%	0.837
Gemcitabine based	13	22.0%	13	17.3%		7	15.2%	8	17.4%	
Platinum based	6	10.2%	8	10.7%		5	10.9%	5	10.9%	

*Tumor location: Intrahepatic stands for intrahepatic cholangiocarcinoma (IHCC), Extrahepatic includes extrahepatic cholangiocarcinoma and gallbladder cancer.

**ECOG PS, Eastern Cooperative Oncology Group performance status.

After matching in a ratio of 1:1, 46 paired patients were enrolled in this study. The balance of the key characteristics was examined, showing that a proper balance between the matched groups was accomplished for all matched characteristics. There were no significant differences between the two matched groups for age, gender, ECOG PS, and primary tumor location, and chemotherapy regimen, number of metastatic organs, liver metastasis, and histology differentiation.

**Figure 2:**
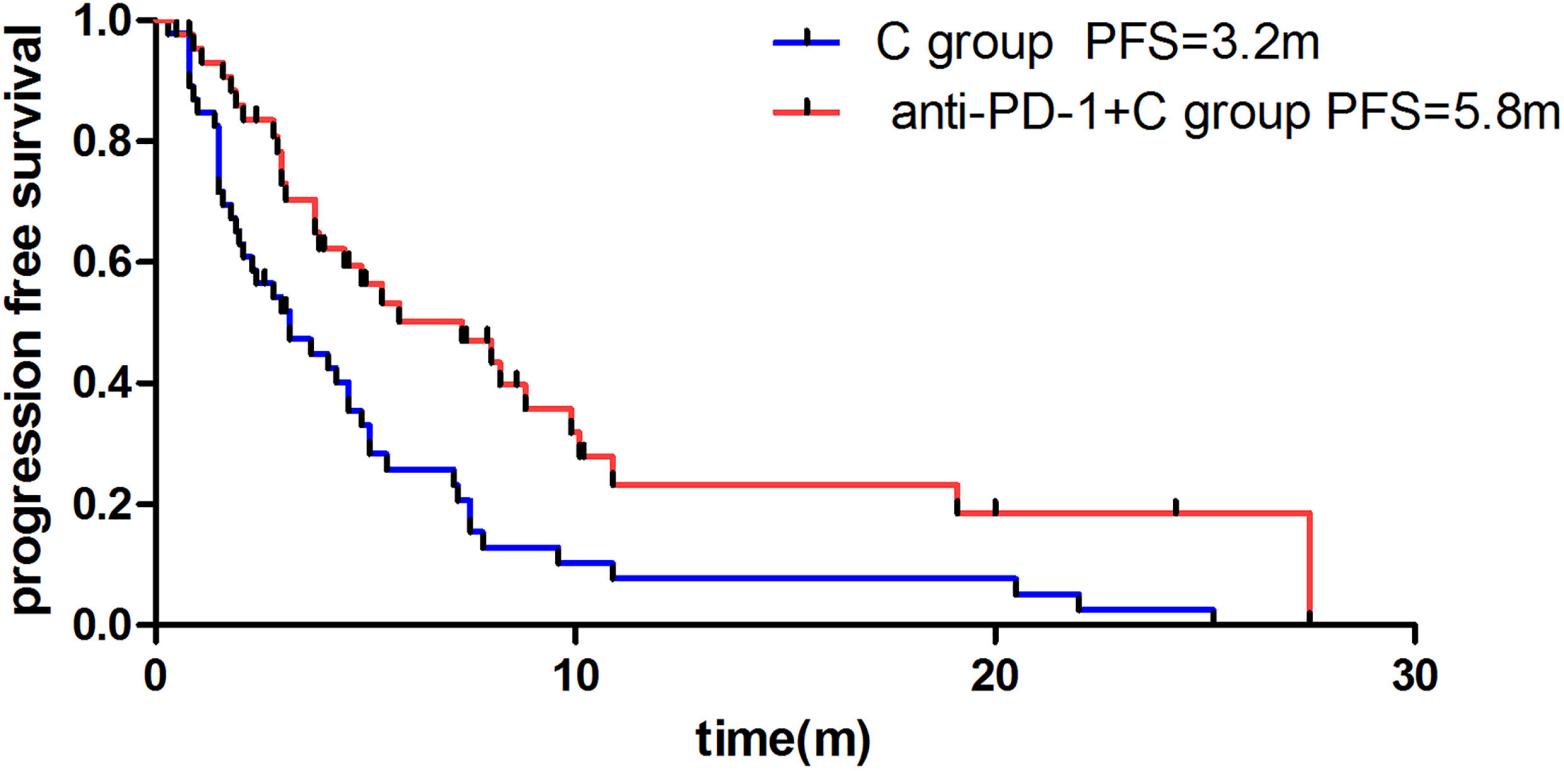
Difference of median progression-free survival of patients in anti-PD-1 +C group and C group.

The median PFS was 5.8m in the anti-PD-1+C group and 3.2m in the C group (HR: 0.47, 95% CI: 0.29 to 0.76, P=0.004) (**Figure 2**). For tumor regression, of the 46 patients in the C group, 7 patients achieved PR, 25 had SD, and 14 had PD. Resulting in an ORR of 15.2% and DCR of 69.6%. Of the 46 patients in the anti-PD-1+C group, 10 patients had PR, 27 had SD, and 9 had PD. The ORR was 21.7% and the DCR was 80.4% (**Table 2**). We found that there was no significant difference in the tumor response (ORR, DCR) between the two groups (P=0.423, P=0.231). We did not obtain the OS due to a failure of follow-up.

**Table 2:** Tumor response for chemotherapy group and chemotherapy +PD-1 inhibitor group after propensity score matching.

Response	C group (N=46)	Anti-PD-1+C group (N=46)
CR		
PR	7	10
SD	25	27
PD	14	9
ORR	15.2%	21.7%
DCR	69.6%	80.4%
Median PFS	3.2m	5.8m

CR,complete response; PR, partial response; SD, stable disease; PD, progressive disease; ORR: CR+PR; DCR: CR+PR+SD.

We further evaluated whether the differences in primary tumor location, liver metastasis, number of metastatic organs could affect the PFS of the two groups (C group and anti-PD-1+C group). In the extrahepatic subgroup, there were no differences in PFS between the two groups (P=0.323), while in the intra-hepatic subgroup the PFS of the anti-PD-1+C group was significantly longer than that of the C group (4.5m vs. 2.4m, P=0.005). For patients with liver metastasis, anti-PD-1+C prolonged PFS compared with chemotherapy alone (8.8m vs. 3.2m, P=0.006). Anti-PD-1+C treatment was more beneficial for patients with less than two metastatic organs (7.3m vs. 3.2m, P=0.008), but not for those with more metastatic organs (4.9m vs. 2.4m, P=0.075).

Adverse events are listed in **Table 3**. The highest non-hematologic grade 1-2 AE was alopecia (41.3% in the C group and 50% in the Anti-PD-1+C group, respectively) and the second highest was peripheral sensory neuropathy (21.7% and 26.1%, respectively). There was a higher incidence of hypothyroidism (13%), rash (10.9%), and fever (13%) in the anti-PD-1+C group. Grade 3 or 4 treatment-related AEs in the anti-PD-1+C group included hypothyroidism (n=3, 6.5%), rash (n=2, 4.3%), and hepatitis (n=1, 2.2%), while only 1 patient had 3-4 grade hepatitis in the C group. There were similar rates of hematologic AEs in the two groups, especially grade 1-2 myelosuppression (32.6% and 41.3%, respectively). No AE-related death was confirmed.

**Table 3:** Analysis of adverse events for chemotherapy group and chemotherapy +PD-1 inhibitor group after propensity score matching.

Adverse Event	No (%)			
	C group		anti-PD-1+C group	
	grade 1-2	grade 3-4	grade 1-2	3-4grade
Non-hematologic				
Rash	2(4.3)	0(0)	5(10.9)	2(4.3)
Nausea and/or vomiting g	5(10.9)	0(0)	6 (13.0)	0(0)
Diarrhea	4(8.6)	0(0)	7(15.2)	0(0)
hepatitis	3(6.5)	1(2.2)	4(8.6)	1(2.2)
Fatigue	5(10.9)	0(0)	7(15.2)	0(0)
Peripheral sensory neuropathy	10(21.7)	0(0)	12(26.1)	0(0)
Fever	2(4.3)	0(0)	6(13.0)	0(0)
Hypothyroidism	1(2.2)	0(0)	6(13.0)	3(6.5)
Alopecia	19(41.3)	0(0)	23(50)	0(0)
Hematologic				
Anemia	5(10.9)	0(0)	6(13.0)	0(0)
Leukopenia	15(32.6)	2(4.3)	19(41.3)	3(6.5)
Thrombocytopenia	5(10.9)	2(4.3)	7(15.2)	1(2.2)

## Discussion

Multiple studies have attempted to improve the low survival rate of patients with BTC. Currently, chemotherapy remains the mainstay treatment strategy for patients with advanced BTC (12). Although the GP regime used as first-line treatment for advanced biliary tract cancer results in a longer PFS and OS, its high toxicity leads to poor tolerance. Therefore, new strategies are required to reduce the toxicity and enhance the benefit of chemotherapy.

Previous studies have shown the promising benefit of anti-PD-1 therapy in many tumor types (13–15) and a good response in patients with ampullary or cholangiocarcinoma harboring the MSI phenotype (7). In addition, chemotherapy is thought to affect the tumor microenvironment and contribute to tumor regression by regulatory T cells (16, 17). Based on those views, it is of interest to explore the efficacy of chemotherapy combined with immunotherapy in advanced BTC.

This retrospective study provides evidence that anti-PD-1 therapy combined with chemotherapy is an effective treatment option for advanced biliary tract cancer. To control for selection bias and confounding factors, the two groups were matched for key variables using propensity score matching. The median PFS of patients treated with PD-1 inhibitor plus chemotherapy was 5.8 months, which was longer than those treated with chemotherapy alone (3.2 months). The hazard ratio was 53% lower in patients who received PD-1 inhibitor plus chemotherapy than those who received chemotherapy alone. Data from a phase III trial of cisplatin plus gemcitabine (GP) versus gemcitabine (G) for biliary tract cancer showed a median progression-free survival of 8.0 months in the cisplatin–gemcitabine group and 5.0 months in the gemcitabine-only group (P<0.001). The PFS rates in our study were numerically not better than the PFS in the above-mentioned trial.

We analyzed why the PFS was notably lower in our study. Firstly, approximately 99.5% of patients in the previous clinical trial were in pretty good condition (EGOG PS score 0-1), while this percentage was 74.7% in our study. Secondly, the clinical trial patients required adequate hematologic and biochemical functions strictly, which is required leniently in real-world clinical applications. Thirdly, the patients in the trial enrolled worldwide, while the patients in our study were national. The PFS in this study is not satisfactory. It still indicates that PD-1 inhibitors enhance the anticancer ability of chemotherapy, which is consistent with studies of PD-1 inhibitor combined therapy applied in other tumor types. For example, the KEYNOTE-189 study (18) presented significantly longer OS and PFS in patients with advanced non-small-cell lung cancer who received Pembrolizumab combined with chemotherapy. In the KEYNOTE-062 study (19) there was a significant increase in the OS of patients with a combined positive score (CPS) >10 who received Pembrolizumab combined with chemotherapy in gastric cancer. Our previous study showed patients with metastatic BTC might benefit more from Nivolumab combined with chemotherapy in the first-line or another line treatment than Nivolumab alone (20). A study from Sun et al. (21) suggested that the combination of PD-1 inhibitors and chemotherapy could provide a significant and clinically-relevant improvement in antitumor activity compared with PD-1 inhibitor monotherapy or chemotherapy alone.

In the present study, no patients achieved a clinical response in either group. Both the ORR and DCR in the anti-PD-1 group are not worse than the average level of other studies of BTC in first-line treatment. We summarized the ORR and DCR from studies for advanced BTC (**Table 4**). As the Table shows, the ORR of Nivolumab plus GP in a phase I study was 37%, with a PFS of 4.2m and OS of 7.9m (9). Tumor regression was not correlated with longer PFS and OS. The highest objective rate reported until now was 46.15% in a phase II study of Camrelizumab (a Chinese anti-PD-1) +FOLFOX4 in BTC (22). We expect the survival time of patients treated with this regimen to improve in the future.

**Table 4:** Summary of current studies of advanced BTC.

Date	Phase	Line	Regime	Case (n)	ORR	DCR	PFS (m)	OS (m)
2011 (12)	III	1	GP	410	13%	81.4%	8.1	11.7
2019 (3)	III	1	GS	177	29.8%		6.8	
JCOG1113								
2017 (8)	I	>1	Pembrolizumab	24	13%	34.8	1.8	6.2
KEYNOTE28								
2019	I	>1	Nivolumab	30	3%	37%	1.4	5.2
(Japan) (9)								
2019	I	1	Nivolumab+GP	30	37%	63%	4.2	7.9
(japan) (9)								
2019ASCO (8)	II	>=1	Pembrolizumab (PD-L1+)	36	11.1%	47.2%	1.5	4.3
2019ASCO (22)	II	1	Camrelizumab+FOLFOX4	47	7.0%	67.4%	–	–
2019ASCO (23)	II	1	SHR-1210+GEMOX	26	46.15%	92.3%	–	–
2019ASCO (24)	II	1	AS	54	27.5%	70.6%	6	13.2

GP means gemcitabine plus cisplatin.

GS was short for gemcitabine plus S-1.

FOLFOX means oxaliplatin combined with calcium folinate and 5-fluorouracil.

GEMOX means Gemcitabine + Oxaliplatin.

AS means nab-paclitaxel plus S-1.

The selection of potential benefit populations for anti-PD-1 combined therapy is critical for patients with advanced BTC. It is thought that the primary tumor location influences the efficacy of overall treatment. A study published in JAMA (25) reported that the median PFS was 12.9 months (95% CI, 8.5-16.1 months) among patients with intrahepatic cholangiocarcinoma (IHCC), 6.0 months (95% CI, 0.7 months to Not Evaluated) among patients with extrahepatic cholangiocarcinoma (EHCC), and 4.1 months (95% CI, 2.1-14.9 months) among patients with gallbladder cancer (GBC) (P = 0.22) when patients were given Gemcitabine, Cisplatin, and nab-Paclitaxel for advanced biliary tract cancers. Klein O, et al. reported that promising efficacy was exclusively observed in patients with intrahepatic cholangiocarcinoma and gallbladder of nivolumab plus ipilimumab Immunotherapy in patients with advanced biliary tract Cancers (26). Our data shows that in the intrahepatic subgroup, patients who received PD-1 inhibitor combined with chemotherapy had longer PFS than those treated with chemotherapy alone. Yet, there was no significant difference in PFS for the two therapy strategies in the extrahepatic subgroup. We also found that PD-1 inhibitor combined with chemotherapy was more beneficial for patients with liver metastasis than chemotherapy alone. This might depend on the microenvironment and more effective T cell infiltration at the tumor site.

PD-L1 expression is a predictive biomarker for the efficacy of anti-PD-1 inhibitors in several tumor types (27, 28). In a keynote series study, it was found to be a common predictor of benefit for populations with esophagus or gastric cancer (29, 30). Instead, in the Checkmate series studies, PD-L1 expression was not correlative with immunotherapy response (31, 32). However, in our study, 10 patients had PD-L1 expression, and 15 patients had PD-L1-negative tumors in the anti-PD-1+C group; for the remainder of patients, the PD-L1 status was unknown. Therefore, PD-L1expression was not enrolled in the analysis.

Hematologic adverse events were similar in the two treatment regimens. There was an increase of grade 3 or 4 rash and hypothyroidism in patients who received PD-1 inhibitors, possibly due to the addition of the PD-1 inhibitor, which was considered an immune-related AE and is consistent with the reports from other studies (33). Immunotherapy may cause immune-related organ dysfunction, including in the lung, skin, thyroid, liver, and kidney (11). The AEs in our study were controllable and there was no AE-related death.

The present study had some limitations. As it was a single-center study, the sample size was small. Therefore, we plan to conduct a multi-center study to demonstrate the added value of pd-1 inhibitor with chemotherapy in patients with advanced BTC.

## Conclusion

This study reveals longer PFS in patients with advanced BTC who receive PD-1 inhibitor combined with a chemotherapy regimen. More studies are needed to identify the potential population who will benefit from this strategy. This study provides valuable clues for a future prospective study.

## Data Availability Statement

The raw data supporting the conclusions of this article will be made available by the authors, without undue reservation.

## Ethics Statement

This study was approved by the ethics committee of PLAGH, and all patients signed consent for making their data public.

## Author Contributions

MG, YZ and TL contributed equally to this work as writer and collect data. GD and NQ as co-corresponding author as supervision. HS, ZW and HY contributed to analysis. All authors contributed to the article and approved the submitted version.

## Funding

This study was funded by the National Natural Science Foundation of China (No. 31671298) and the National Key Research and Development Program of China (2017YFC1308902).

## Conflict of Interest

The authors declare that the research was conducted in the absence of any commercial or financial relationships that could be construed as a potential conflict of interest.
